# Medical Lectures

**Published:** 1844-12

**Authors:** 


*Medical Lectures*—In St. Louis University, begin in November. Although there are two schools of medicine in Missouri, both in the thriving city of St. Louis, they have sufficient support to warrant them in looking forward with high expectations of usefulness and distinction in future years. Of the University School of Medicine, very little need be said of it to show how strong a hold it has on the public confidence at the West. Nine students received doctorates at the late commencement.

In the *College of Dental Surgery*, at Baltimore, the lectures begin in November. At one time there was a disposition on the part of some gentlemen to throw a shade of doubt on the success of this school. They could not believe that there were young men enough in the world, who wished to become scientific dentists, to support an institution organized on the principles of the Baltimore College. It has so far, however, lived down all opposition, and disappointed those who prophesied its downfall. Seventeen gentlemen had received degrees at the close of the last session—*Doctors of Dental Surgery*. There may be a host frowning upon this College, and foretelling its ultimate neglect; but they cannot alter its destiny. It is a school for making safe dentists, and the people will have confidence in those who have been taught there.—*Boston Med. & Surg. Journal.*

